# GABA_A_ Receptor-Mediated Epileptogenicity in Focal Cortical Dysplasia (FCD) Depends on Age at Epilepsy Onset

**DOI:** 10.3389/fncel.2020.562811

**Published:** 2020-09-30

**Authors:** Jyotirmoy Banerjee, Soumil Dey, Aparna Banerjee Dixit, Ramesh Doddamani, Meher Chand Sharma, Ajay Garg, P. Sarat Chandra, Manjari Tripathi

**Affiliations:** ^1^Department of Biophysics, All India Institute of Medical Sciences, New Delhi, India; ^2^Department of Neurosurgery, All India Institute of Medical Sciences, New Delhi, India; ^3^Dr. B R Ambedkar Centre for Biomedical Research, University of Delhi, New Delhi, India; ^4^Department of Neuropathology, All India Institute of Medical Sciences, New Delhi, India; ^5^Department of Neuroimaging and Interventional Neuroradiology, All India Institute of Medical Sciences, New Delhi, India; ^6^Department of Neurology, All India Institute of Medical Sciences, New Delhi, India

**Keywords:** drug-resistant epilepsy, GABAergic activity, electrocorticography (ECoG), resected brain sample, patch-clamp technique

## Abstract

Enhanced spontaneous GABA_A_ receptor activity is associated with focal cortical dysplasia (FCD), a developmental malformation of the cerebral cortex. Clinical manifestations in FCD vary with age at epilepsy onset with a more favorable prognosis in patients with late-onset (LO) compared to that in cases with early-onset (EO). This study was designed to test the hypothesis in FCD that spontaneous GABA_A_ receptor-mediated epileptogenicity depends on the age at epilepsy onset and varies between patients with early and late-onset age in FCD. To this end, brain specimens were obtained from the maximal spiking region (MAX) and minimal spiking region (MIN) of the epileptic foci of EO (*n* = 14, mean age = 10.6 ± 2.9 years) and LO (*n* = 10, mean age = 27 ± 5.6 years) patients undergoing electrocorticography (ECoG) guided surgery. The whole-cell patch-clamp technique was used to record spontaneous GABAergic currents from normal-looking pyramidal neurons in slice preparations of resected brain samples. We detected higher frequency and amplitude of GABAergic events in MAX samples compared to MIN samples of LO patients, while they were comparable in MIN and MAX samples of EO patients. Further GABAergic activity in the MIN and MAX samples of EO patients was higher than the MIN samples of LO patients. This suggests that in LO patients, GABA_A_ receptor-mediated epileptogenicity is confined only to the high spiking areas, but in EO patients, it affects low spiking regions as well.

## Introduction

Focal cortical dysplasia (FCD) is a developmental disorder and the most common substrate of pediatric drug-resistant epilepsy (DRE). We have earlier shown that surgical management of pediatric patients with DRE helped in reducing the seizure frequency (Dwivedi et al., 2017; Chandra et al., 2018). Assessing the epileptogenic zone is paramount for resective surgery, especially in FCDs. Failure to obtain seizure freedom in some patients with FCD even after surgical resection could be due to an inaccurate quantification of epileptogenicity, which may have extended beyond the lesion (Aubert et al., 2009). FCD is a malformation of cortical development but, features such as age at epilepsy onset in this pathology are poorly understood. Epileptogenicity and severity of manifestations in FCD may vary with age at epilepsy onset (Fauser et al., 2006), with early-onset generally being an unfavorable prognostic factor and a more favorable prognosis in patients with late-onset (Bartolomei et al., 1999). Siegel et al. (2005), in their study involving 213 patients with FCD, have shown that patients with epilepsy onset beyond the age of 18 years accounted for 10% of patients with FCD IIa and b. Further, Fauser et al. (2006) have shown adult-onset in 6.5% of the total number of patients and 4.2% in patients with FCD IIa and b in their study involving 120 patients with FCD. These findings indicate that structural abnormalities in the cortex of patients with FCD with abnormal cells can be silent over the decades.

Immature neuronal networks in FCD lead to altered GABA_A_ receptor function thereby causing abnormal synaptic transmission (Cherubini et al., 1991; Cepeda et al., 2005). Brain specimens obtained from patients with FCD type I and II retain their epileptogenicity, which is dependent on enhanced spontaneous interictal discharges mediated by GABA_A_ receptors (Blauwblomme et al., 2019). To our knowledge, so far, no study has focussed on age at epilepsy onset and GABA_A_ receptor-mediated epileptogenicity in patients with FCD. Studies on GABAergic activity in resected brain specimens obtained only from pediatric patients with FCD could be biased concerning the age of epilepsy onset. We analyzed the spontaneous GABAergic activity in acute slice preparations of resected brain specimens, electro-corticographically (ECoG) graded as high and low spiking (Mathern et al., 2000; Tripathi et al., 2010) obtained from patients with FCD type I and II having early (EO) and late (LO) age at epilepsy onset.

## Materials and Methods

### Ethics Statement

The study was carried out following the recommendations of the institutional ethics committee (IEC), All India Institute of Medical Sciences, New Delhi, India. Written, informed consent was obtained for all the patients.

### Patients

Twenty-four patients with FCD type I and II, confirmed by histopathology (Supplementary Figure S1), who underwent resective surgery from 2015 to 2019 were included in this study (Table 1). Patients with dual pathology like temporal lobe epilepsy were not included in this study. Patients were grouped according to age at onset of epilepsy (Bartolomei et al., 1999). The EO group included 14 patients in whom the first seizure occurred before the age of 12 and the LO group included 10 patients in whom the first seizure occurred after the age of 12. The mean age of the EO group was 10.6 ± 2.9 years and the mean age for the LO group was 27 ± 5.6 years. Pre-surgical evaluations included video EEG, MRI, FDG-PET, and ECoG. Based on ECoG recordings, the regions were graded from scores 1–5 (Mathern et al., 2000; Tripathi et al., 2010), with grade 3 and above as a highly spiking zone (MAX) and grade 1 as low spiking zone (MIN). Representative ECoG traces from MIN and MAX regions of patients with FCD are shown in Supplementary Figure S2. Surgical resection of ECoG graded cortical samples was performed as per the previously reported protocol (Tripathi et al., 2010; Dwivedi et al., 2017).

**Table 1:** Clinical characteristics of patients selected for the study.

Patient ID*	Sex	Age (years)	Age at epilepsy onset	AEDs at the time of surgery	ECoG grading (Mathern et al., 2000; Tripathi et al., 2010)	Histology	Size	Brain location	Engel surgical outcome scale (Engel, 1983)
**FCD patients with early age at epilepsy onset (EO)**									
EO1	M	9	1 year 6 months	CBZ, CLM, PHY, VAL	MAX-4 MIN-1	FCD IIa	Multilobar	Right temporal and frontal cortex	I
EO2	M	10	4 years	CLM, LCS, LVM, VAL	MAX-4 MIN-1	FCD IIa	Unilobar	Left cingulate gyrus	I
EO3	F	12	5 years	LVM, OXC, CLM	MAX-3 MIN-1	FCD IIb	Unilobar	Right frontal inferior gyrus	I
EO4	F	6	4 months	CLM, OXCBZ	MAX-3 MIN-1	FCD Ic	Multilobar	Right fronto-temporal	I
EO5	M	12	2 years	LMG, SV, TPM	MAX-5 MIN-1	FCD Ic	Unilobar	Anterior frontal cortex	I
EO6	M	10	2 years 6 months	CLM, LVM, TPM	MAX-3 MIN-1	FCD IIa	Multilobar	Opercular, insular, and anterior temporal cortex	I
EO7	M	11	7 months	LMG, CLM, VAL	MAX-3 MIN-1	FCD Ic	Unilobar	Right basifrontal cortex	I
EO8	F	12	6 years	LVM, CBZ, TPM, CLM	MAX-3 MIN-1	FCD IIa	Multilobar	Left temporoparieto-occipital	I
EO9	M	13	2 years	TPM, CLM, LCM, LVM VAL	MAX-3 MIN-1	FCD Ic	Multilobar	Right Temporal and insular dysplasia	I
EO10	F	3	5 months	CBZ, CLM, LVM, ZNS	MAX-3 MIN-1	FCD Ic	Unilobar	Left anterior frontal cortex	I
EO11	F	3	5 months	CBZ, LCS, LMG, L VM	MAX-3 MIN-1	FCD Ib	Multilobar	Right perisylvian region predominantly involving the anterior and medial region	I
EO12	M	12	5 years	CLM, CBZ, LVM	MAX-3 MIN-1	FCD IIa	Unilobar	Left anterior superior frontal and cingulate gyrus	I
EO13	M	13	2 years	TPM, CLM, LCM, LVM, VAL	MAX-3 MIN-1	FCD Ib	Unilobar	Posterior segment of superior frontal gyrus anterior to premotor cortex	I
EO14	F	13	8 months	LVM, LCM, VAL, CLM, CBZ	MAX-3 MIN-1	FCD Ic	Unilobar	Left antrior superior frontal gyrus	I
**FCD patients with late age at epilepsy onset (LO)**									
LO1	M	22	15 years	LVM, LCM, LMG, VAL	MAX-3 MIN-1	FCD Ic	Unilobar	Right frontal cortex	I
LO2	F	29	15 years	LVM, CBZ, CLM	MAX-5 MIN-1	FCD Ic	Unilobar	Left lateral temporal and basitemporal cortex	I
LO3	F	22	16 years	CLM, LVM, SV	MAX-5 MIN-1	FCD IIb	Mutilobar	Bottom of the sulcus and lateral margin of the left lateral ventricle.	I
LO4	M	36	19 years	CLM, LVM	MAX-3 MIN-1	FCD IIa	Unilobar	Left amygdalohippocampal dysplasia	I
LO5	F	24	20 years	CLM, LVM, TPM	MAX-5 MIN-1	FCD Ib	Unilobar	Right posterior temporal cortex	I
LO6	M	31	15 years	CLM, LVM, OXC, LMG	MAX-3 MIN-1	FCD Ic	Unilobar	Superior frontal gyrus	I
LO7	M	24	14 years	VAL, PHE, CLM	MAX-4 MIN-1	FCD Ic	Unilobar	Left posterior temporal cortical dysplasia	I
LO8	M	36	20 years	VAL, LVM, CBZ	MAX-3 MIN-1	FCD Ib	Unilobar	Right middle frontal gyrus	I
LO9	M	21	15 years	CLM, LVM	MAX-4 MIN-1	FCD IIa	Unilobar	Right anterior temporal cortical dysplasia	I
LO10	F	25	16 years	LVM, CBZ, CLM, VAL	MAX-4 MIN-1	FCD IIa	Unilobar	Dysplasia in right posterior temporal region	I

*EO1-EO14 = FCD Patients with early age at epilepsy onset (<12 years of age at epilepsy onset). LO1-EO10, FCD Patients with late age at epilepsy onset (≥12 years of age at epilepsy onset). AED, Antiepileptic drug; M, Male; F, Female; CBZ, Carbamazepine; OXC, Oxcarbazepine; CLM, Clobazam; VAL, Valproic Acid; PHE, Phenytoin; LVM, Levetiracetam; LMG, Lamotrigine; TPM, Topiramate; PHB, Phenobarbital; LCS, Lacosamide; ZNS, Zonisamide*.

### *In vitro* Electrophysiology

Resected brain samples obtained from patients with FCD were collected in well carbogenated, ice-cold artificial cerebrospinal fluid (ACSF) composed of 2 mM CaCl_2_, 25 mM NaHCO_3_, 1.25 mM NaH_2_PO_4_, 125 mM NaCl, 2.5 mM KCl, 1 mM MgCl_2_, and 25 mM Glucose. 350–400 μm thick slices were prepared using a vibratome (VT1000S, Leica Biosystems) and incubated at room temperature for at least 30 mins. Slices were prepared by making tangential cuts to the outer surface of the cortical specimens and were stored at room temperature in ACSF, constantly bubbled with 95% O_2_ and 5% CO_2_. Infrared-assisted video-microscopy with differential interference contrast (IR-DIC) was used to morphologically identify pyramidal neurons located in slice preparations. Normal appearing pyramidal neurons were morphologically identified by its pyramid-like and a single thick tapering apical dendrite. Cells on the surface slice preparations were usually dead, so we used the pale-looking pyramidal neurons from layer 3 or 4 for our studies. Passive membrane properties of neurons on FCD samples were determined by using membrane test function integrated into pCLAMP 10 software (Molecular Devices, Sunnyvale, CA, USA). Whole-cell recordings were obtained from the soma of visually-identified pyramidal neurons in slice preparations using an Axopatch 200B amplifier (Molecular Devices, San Jose, CA, USA). Whole-cell mode of the patch-clamp technique was used to record spontaneous inhibitory postsynaptic currents (sIPSCs) at 0 mV and spontaneous excitatory postsynaptic currents (sEPSCs) at −70 mV from normal-appearing pyramidal neurons in slice preparations (Banerjee et al., 2017) using an Axopatch 200B amplifier (Molecular Devices, San Jose, CA, USA). sIPSCs were completely blocked by treatment with ACSF containing bicuculline (10 μM) and the sEPSCs were completely blocked by treatment with ACSF-containing admixture of CNQX (10 μM) and APV (50 μM; data not shown). Miniature IPSCs (mIPSCs) were recorded in the presence of 200 nM tetrodotoxin (TTX). P-97 Flaming-Brown puller (Sutter Instruments, Novato, CA, USA) was used to pull patch pipettes from borosilicate glass capillaries (OD 1.2 mm, World Precision Instruments, New Haven, CT, USA). Patch pipettes were filled with internal solution containing 10 mM HEPES, 130 mM Cs-methanesulfonate, 10 mM EGTA, 10 mM CsCl, 2 mM MgCl_2_ and 5 mM QX-314 (Alkondon et al., 2000). The resistance of the filled patch pipettes was in the range of 4–6 MΩ. The access resistance during each recording ranged between 10 and 20 MΩ and if it increased by more than 20% from initial values, data from those neurons were not considered for analysis. All the recordings were performed at room temperature (Alkondon et al., 2000). For experiments involving the measurement of sIPSCs, we have recorded from 14 neurons each from both MIN and MAX samples of 14 patients with EO. While in the case of LO, we have recorded the sIPSCs from 10 neurons each from both MIN and MAX samples of 10 patients with LO. For the measurement of mIPSCs, we used six neurons each from MIN and MAX samples of six patients with EO. In the case of LO, we used five neurons from both MIN and MAX samples obtained from patients with LO. For the measurement of the index ratio of the frequency of GABAergic/glutamatergic ratio, we used five neurons each from MIN and MAX samples obtained from LO patients and six neurons each from MIN and MAX samples in the case of EO patients.

### Data Analysis and Statistics

sIPSCs/sEPSCs and mIPSCs were analyzed in 5-min recordings using pCLAMP 10.0 software (Molecular Devices, Sunnyvale, CA, USA). Frequency, peak amplitude, rise time (10–90%), and decay-time constant (τ_d_) of the IPSCs and EPSCs were measured. The threshold amplitude for detecting sIPSCs was set at 10 pA and for sEPSCs at 5 pA. sIPSCs and sEPSCs detected by the software were visually inspected to minimize errors. Events that did not show a typical synaptic waveform were rejected manually. Events which showed a typical synaptic waveform with a sharp rising phase and an exponential decay were identified manually and used for kinetic analysis. Double- and multiple-peak currents were excluded for the determination of PSC properties but included for calculation of frequency of PSCs. Rise times and decay time constant (τ_d_) were determined during the analysis of the averaged chosen single events aligned at half rise time. Data are expressed as mean ± SEM of results obtained from various groups and statistical significance was analyzed using one-way ANOVA in Sigmaplot 14.0 (Systat Software, Inc., Chicago, IL, USA). The cumulative distributions of events in EO vs. LO groups were compared using the Kolmogorov–Smirnov test (K–S test). Data of postsynaptic events recorded from neurons in EO and LO groups were pooled together and subjected to the K–S test using the Clampfit module of the pCLAMP 10.0 software.

## Results

**Figure 1:**
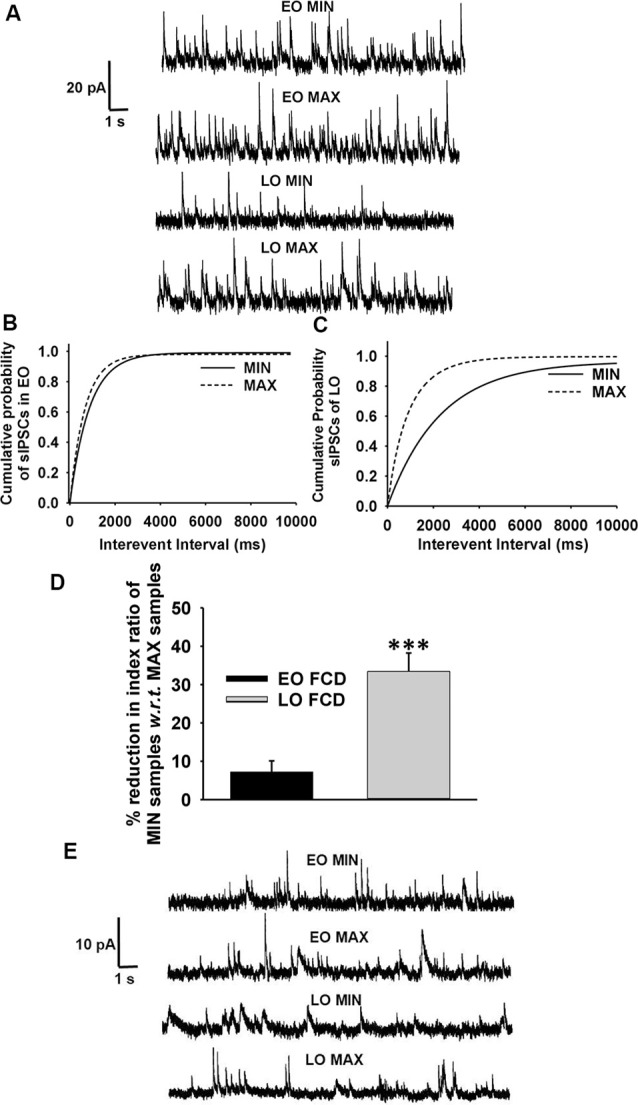
GABAergic activity in low and high spiking zones of patients with focal cortical dysplasia (FCD) varies with age at epilepsy onset. **(A)** Sample recordings of spontaneous GABAergic postsynaptic currents (PSCs) at 0 mV were obtained from pyramidal neurons of early-onset minimal spiking region (EO MIN), early-onset maximal spiking region (EO MAX), late-onset minimal spiking region (LO MIN), and late-onset maximal spiking region (LO MAX) samples. **(B)** Plots show the cumulative distribution of inter-event intervals of spontaneous IPSCs (sIPSCs) recorded from pyramidal neurons of EO MIN and EO MAX samples. Plots represent data from 14 neurons each from 14 MIN and MAX samples obtained from EO patients. There was no significant change in the cumulative distribution of inter-event intervals in EO MAX and EO MIN samples. **(C)** Plots show the cumulative distribution of inter-event intervals of sIPSCs recorded from pyramidal neurons of LO MIN and LO MAX samples. Plots represent data from 10 neurons each from 10 MIN and MAX samples obtained from LO patients. There was significant leftward displacement of the cumulative distribution of inter-event intervals in the case of LO MAX samples compared to that in LO MIN samples [*p* < 0.01 according to Kolmogorov–Smirnov test (K–S) test]. **(D)** Plots showing percent reduction in the index ratio of MIN samples of EO and LO patients *w.r.t*. that in their respective MAX samples. Reduction in the index ratio in MIN samples of EO patients (*n* = 6) *w.r.t.* MAX samples were significantly lower than that in the case of LO patients (*n* = 5). ***p < 0.001 compared to the LO group according to one-way ANOVA followed by Dunnett *post hoc* test. **(E)** Representative traces of mIPSCs recorded at 0 mV from pyramidal neurons of MIN and MAX samples of EO and LO patients after superfusion of slices with action-potential inhibitor tetrodotoxin (TTX; 200 nM) for 10 min.

Passive membrane properties of pyramidal neurons were measured in samples obtained from patients with FCD. The cell capacitance and input resistance of pyramidal neurons in FCD samples were 178 ± 14 pF and 174 ± 21 MΩ, respectively. Bicuculline-sensitive spontaneous inhibitory postsynaptic currents (sIPSCs) were recorded from pyramidal neurons in the brain specimens obtained from EO and LO patients with FCD (Figure 1A). Quantitative analysis (Figures 1B,C) showed no change in the cumulative distribution of inter-event interval of sIPSCs of MIN (*n* = 14) and MAX (*n* = 14) samples of EO patients while there was a leftward shift in the cumulative distribution of inter-event interval of MAX samples compared to that in MIN samples of patients with LO (*n* = 10). In the case of EO patients, mean peak amplitude and frequency of sIPSCs in the MIN sample were comparable to that in MAX samples, but higher than MIN samples of LO patients. The mean peak amplitude and frequency of sIPSCs in MIN samples were significantly lower than those in MAX samples in LO patients (Table 2).

**Table 2:** Characteristics of sIPSCs and mIPSCs: kinetic parameters of sIPSC and mIPSC recorded from pyramidal neurons in EO MAX, EO MIN, LO MAX, and LO MIN samples.

Patient	Frequency (Hz)	Amplitude (pA)	Rise time 10–90% (ms)	τd (ms)
sIPSC
EO MAX	3.2 ± 0.3^@^	28.2 ± 3.1**^@^	2.0 ± 0.12	45.4 ± 4.2**
EO MIN	2.9 ± 0.2^@^	26.6 ± 2.1**^@^	1.8 ± 0.24	43.1 ± 3.1**
LO MAX	2.8 ± 0.2**	25.7 ± 3.8**	1.9 ± 0.2	44.3 ± 3.9**
LO MIN	2.0 ± 0.1	19.2 ± 1.7^#^	2.1 ± 0.2	38.6 ± 4.6
mIPSC
EO MAX	1.23 ± 0.3**^@^	15.8 ± 2.0**^@^	1.82 ± 0.2	41.2 ± 3.2**
EO MIN	1.12 ± 0.2**^@^	14.5 ± 1.7**^@^	1.75 ± 0.1	40.3 ± 2.8**
LO MAX	1.08 ± 0.2	14.4 ± 2.3**	1.85 ± 0.2	39.4 ± 2.6**
LO MIN	0.81 ± 0.1**^,#^	11.6 ± 1.9^#^	1.80 ± 0.2	34.1 ± 3.5^#^

Data are presented as mean ± SEM of results obtained from as many numbers of neurons and patients as mentioned in the Figure 1. **p < 0.01 compared to respective MIN samples according to one-way ANOVA followed by Dunnett *post hoc* test. ^#^*p* < 0.01 compared to LO MAX samples according to one-way ANOVA followed by Dunnett *post hoc* test. ^@^*p* < 0.01 compared to LO MIN samples according to one-way ANOVA followed by Dunnett *post hoc* test.

Spontaneous EPSCs (sEPSCs) recorded at −70 mV from MIN and MAX samples of both EO and LO patients with FCD, appeared as inward events (Supplementary Figure S3) sensitive to CNQX (10 μM), AMPA receptor antagonist, in admixture with the APV (50 μM), NMDA receptor antagonist (data not shown). The average frequency of sEPSCs in EO MIN (*n* = 6), EO MAX (*n* = 6), LO MIN (*n* = 5) and LO MAX (*n* = 5) samples were 0.58 ± 0.09 Hz, 0.63 ± 0.08 Hz, 0.57 ± 0.07 Hz and 0.62 ± 0.09 Hz, respectively. The average frequency of glutamatergic events in MIN and MAX samples of both LO and EO patients were comparable. Further, an index ratio was obtained by dividing the average frequency of GABAergic events by that of glutamatergic events in a subset of neurons where the sIPSCs and sEPSCs could be recorded in the same cell. The index ratio of the frequency of spontaneous GABAergic/glutamatergic activity in MIN region samples was lower (3.2 ± 0.7) compared to that in MAX region samples (4.8 ± 0.9) of LO patients (*n* = 5). While in EO patients (*n* = 6) it was comparable in the MIN (4.6 ± 0.8) and the MAX (4.9 ± 0.9) samples suggesting a differential GABAergic activity in the MIN and MAX samples of the two groups (Figure 1D).

To measure the changes in action potential-independent GABAergic events in samples obtained from patients with EO and LO, slice preparations were superfused with TTX (200 nM)-containing ACSF and miniature inhibitory postsynaptic currents (mIPSCs) were recorded (Figure 1E). The frequency and amplitude of mIPSCs in samples obtained from the MIN (*n* = 6) region were comparable to that in the MAX (*n* = 6) region of patients with EO but higher than that in the case of MIN (*n* = 5) samples of LO patients (Table 2). The frequency and amplitude of mIPSCs in MIN (*n* = 5) samples were significantly lower than MAX (*n* = 5) samples in LO patients. The decay time constant (τ_d_) of sIPSCs and mIPSCs in MIN and MAX samples of EO patients was comparable, but in the case of LO, it was prolonged in MAX samples compared to the MIN samples (Table 2). Rise time values of sIPSCs and mIPSCs (Table 2) of MIN and MAX samples of both EO and LO patients were comparable. Further, we also compared the characteristics of sIPSCs and mIPSCs between the MIN and MAX samples of patients with FCD, without taking the age of onset into account. We observed a significant increase in the frequency and amplitude, of both sIPSCs and mIPSCs, in the MAX samples as compared to the MIN samples of patients with FCD while the rise time and τ_d_ remained comparable in the two regions (Supplementary Table S1).

## Discussion

Normal appearing pyramidal neurons in the epileptic foci of patients with FCD retain immature GABAergic inputs responsible for developing dysmature neuronal network (Cherubini et al., 1991; Cepeda et al., 2007, 2014). In FCD type I and II, network-level changes lead to GABA_A_ receptor-mediated spontaneous interictal discharges (Blauwblomme et al., 2019). Dysfunctional synaptic inhibition has been shown in pyramidal neurons of resected brain samples obtained from adult patients with FCD type II as compared to the samples obtained from patients with mesial temporal lobe sclerosis (Calcagnotto et al., 2005). Andre et al. (2010) recorded spontaneous GABAergic activity in pyramidal neurons of samples obtained from patients with FCD type I and II and observed that the frequency and amplitude of sIPSCs were higher only in the case of FCD type II, and not in FCD type I, compared with that in a non-FCD pathology. It has been reported that in normal pyramidal neurons of brain samples obtained from pediatric patients with FCD type II the frequency and amplitude of sIPSCs are increased and vary between various cell types (Cepeda et al., 2012). In our study difference in the amplitude of sIPSCs and mIPSCs between MIN and MAX in LO indicates enhanced synaptic transmission through increased GABA_A_ receptor density on the postsynaptic membrane of neurons in the MAX samples compared to MIN samples. In LO, prolonged decay time constants of sIPSCs and mIPSCs and a predominant GABAergic synaptic input onto pyramidal neurons as represented by higher GABAergic/Glutamatergic index ratio in the MAX samples compared to MIN samples indicated reinforced GABA_A_ receptor activity under basal conditions, but only in the severely spiking areas. It has been demonstrated in pediatric patients with FCD that GABA_A_ receptor-mediated synaptic activity is increased compared with glutamatergic activity (Cepeda et al., 2005) and in some cases, GABA is depolarizing (Cepeda et al., 2007). In the case of EO, the mean peak amplitude, τ_d_ and the index ratio were comparable between MIN and MAX samples but were significantly higher than that in the MIN samples of LO (Table 2). This indicates higher GABA_A_ receptor-mediated epileptogenicity in both MIN and MAX regions of EO, but only in the MAX region in LO patients.

Alteration in the number of GABAergic neurons and action potential-independent release of GABA from these terminals regulate the frequency of sIPSCs and mIPSCs (Figure 1; Table 2; Supplementary Table S1). Possibly, in LO patients the network of GABAergic neurons is altered to induce a greater number of synapses or greater quantal release in the MAX region compared to the MIN region indicating a robust GABA_A_ receptor-mediated epileptiform synchronization only in the high spiking region (D’Antuono et al., 2004). While in the case of EO patients quantal release of GABA on to the pyramidal neurons may be similar in both high and low spiking zones, but higher than the MIN region of LO patients. An increase in the release of GABA further indicates immaturity of neuronal circuits associated with FCD, where GABA may act as an excitatory neurotransmitter (Abdijadid et al., 2015). It has been reported that decreased expression of potassium-chloride cotransporter 2 (KCC2) expression and enhanced activity of sodium-potassium-chloride cotransporter 1 (NKCC1) contributes to altered neuronal chloride regulation in FCD (Blauwblomme et al., 2019). Increased NKCC1 activity, which drives neuronal chloride influx causing the depolarizing effect of GABA, may not be counterbalanced by KCC2 due to its lower expression in FCD. This could lead to paradoxical depolarization of pyramidal neurons due to altered intracellular chloride concentration causing enhanced spontaneous GABA_A_ receptor-mediated interictal epileptogenicity in patients with FCD. The spatial distribution of the GABA_A_ receptors on the neurons remained unaltered in MIN and MAX samples of both EO and LO patients, as depicted by the rise time values of sIPSCs and mIPSCs. Further, a comparison of characteristics of sIPSCs and mIPSCs in the MIN and MAX region samples obtained from patients with FCD regardless of the age of onset of patients showed that the frequency and amplitude of sIPSCs and mIPSCs in MAX samples were higher than that in the case of MIN samples (Supplementary Table S1). In our study, we did not observe any correlation between age at epilepsy onset and GABAergic activity in patients FCD, in both LO and EO groups, concerning the brain localization of the resected specimens. Earlier, Fauser et al. (2006) also reported that in their study the age of onset in temporal, extratemporal, and multilobar FCD was similar. Lortie et al. (2002) also could not find a correlation between age of onset and the topography of the lesion in patients with FCD.

Our study demonstrates that under resting conditions epileptogenicity mediated by altered GABAergic network is dependent on the age at epilepsy onset in patients with FCD, with GABA_A_ receptor-mediated epileptogenicity affecting even low spiking areas in case of patients with early-onset. C-flumazenil-positron emission tomography studies showed an abnormality in GABA_A_ receptors even in regions distant from primary focus in patients with FCD (Juhász et al., 2009). Differential GABA_A_ receptor-mediated epileptogenicity could explain the favorable manifestations and prognosis in patients with LO than in EO cases (Bartolomei et al., 1999). Increased spontaneous GABAergic activity and pacemaker GABA synaptic activity has been shown in samples with pathological high-frequency oscillations (HFOs) obtained from patients with FCD (Cepeda et al., 2014, 2019). It has been recently shown that epileptiform synchronization, mediated by 4-aminopyridine (4AP)-containing medium, leading to *in vitro* ictal activity in the human FCD tissues may be facilitated by the decreased ability of GABA_B_ receptors to control GABA release from interneuron terminals (D’Antuono et al., 2004; Levinson et al., 2020). Thus, it may be possible that such an increase in spontaneous GABA_A_ receptor activity could be due to the altered function of presynaptic GABA_B_ receptors. The possible contribution of GABA_B_ receptor function to differential spontaneous GABAergic activity in the EO and LO patients with FCD needs to be further investigated.

Baldassari et al. (2019) have reported a genotype-phenotype relationship and observed a significant difference in the distribution of the age at seizure onset among mild MCDs (mMCD) and FCD patients with somatic variants in PIK3CA, AKT3, RHEB, MTOR, TSC1/2, and SLC35A2 or germline variants in TSC2 or DEPDC5, reflecting a link between the occurrence of these variants and early onset. In a previous study, we have shown that DNA methylation regulated potential gene networks, pathophysiological pathways including PDGFR, EGFR, NTRK3(RTKs) as well as mTOR signaling and several other potential epilepsy-related genes associated with FCD type II (Dixit et al., 2018). It may be possible that genes involved in PDGFR, EGFR, NTRK3, and mTOR signaling and/or genetic variations in PIK3CA, AKT3, RHEB, MTOR, TSC1/2, SLC35A2, TSC2, DEPDC5 genes may have contributed to the differential regulation of GABAergic activity in EO and LO patients with FCD. Additional Next-Gen sequencing (NGS)-based studies on large cohorts of FCD type I and II patients, in a subtype-specific manner, are needed to identify various potential molecules, followed by specific inhibitor-based studies to understand a correlation between age at epilepsy onset and the regulation of GABA_A_ receptor activity.

This human study has a few limitations. First, the patients with FCD were on a combination of anti-epileptic drugs which may affect GABA_A_ receptor activity as well as the ECoG spikes. Second, it was not possible to obtain age-matched patients for the EO and LO groups. Third, the sample size was small with 14 patients in the EO group and 10 patients in the LO group. Moreover, our study involved different FCD subtypes and the samples were collected from different cortical regions. Also, the contribution of circadian variations (Karoly et al., 2016) to ECoG spikes could not be ruled out in defining the low and high spiking zones in our study.

## Conclusion

This is the first direct evidence, at the cellular level, to show that under resting conditions GABAergic signals in FCD vary between low spiking and high spiking regions of patients with early and late age at epilepsy onset. This small exploratory study shows a tight association between GABA_A_ receptor function and age at epilepsy onset in patients with FCD. Studies comparing GABAergic activity in samples obtained from MIN and MAX regions of all FCD subtypes on larger sample sizes are warranted.

## Data Availability Statement

All datasets presented in this study are included in the article/Supplementary Material.

## Ethics Statement

The studies involving human participants were reviewed and approved by Institutional ethics committee (IEC), All India Institute of Medical Sciences, New Delhi, India. Written informed consent to participate in this study was provided by the participants’ legal guardian/next of kin. Written informed consent was obtained from the individual(s), and minor(s)’ legal guardian/next of kin, for the publication of any potentially identifiable images or data included in this article.

## Author Contributions

JB, AD, PC, and MT contributed to the conception and design of the study. JB, SD, RD, MS, AG, PC, and MT contributed to the acquisition and analysis of data. JB, AD, PC, and MT contributed to drafting the text and preparing the figures. All authors contributed to the article and approved the submitted version.

## Conflict of Interest

The authors declare that the research was conducted in the absence of any commercial or financial relationships that could be construed as a potential conflict of interest.

## Supplementary Material

FIGURE S1 Histology revealed abnormal cytoarchitecture in resected brain specimens obtained from patients with focal cortical dysplasia (FCD). **(A)** H&E staining of FCD type I sections showing loss of laminar architecture of the cerebral cortex, the haphazard arrangement of the neurons (×400). **(B)** NeuN immunohistochemistry shows abnormal neurons express immunopositivity for anti-NeuN antibody (magnification 400×). **(C)** H&E staining of FCD type IIa sections showing loss of laminar architecture (×400). **(D)** CD34 immunohistochemistry shows some of these clustered abnormal neurons express bush like immunopositivity For CD34 (magnification 400×). **(E,F)** H&E staining of FCD type IIb sections showing neuromegaly, loss of laminar architecture and larger neurons with abundant eosinophilic cytoplasm (glassy neurons; magnification 400×).

FIGURE S2 Representative electrocorticography (ECoG) of a 13-year-old male patient with early-onset (EO) FCD. **(A)** Top left panel displays MRI brain axial section FLAIR sequence showing right posterior superior temporal gyrus bottom of sulcus dysplasia. Intraoperative picture (bottom left panel) showing a 4 × 5 ECoG grid placed on the temporoparietal region. The right panel shows preoperative ECoG recording (sensitivity-300 μv/cm & high cut-70 Hz) with poly spike bursts beginning in the leads 8 and 5 (red ellipse) to involve all the leads producing multiple brief burst like pattern (red rectangles) against a fast background representing grade 3 (Mathern et al., 2000; Tripathi et al., 2010). **(B)** ECoG grid was placed over the resection cavity, following the first trans-sulcal resection of the dysplasia (left panel). ECoG tracings (right panel) showing persistent spikes (grade 3) occurring in brief bursts (black rectangles). **(C)** ECoG recording following the extension of resection (second resection) to the overlying temporal and partly parietal cortex surrounding the previous resection cavity. The ECoG tracing improved from grade-3 to grade-2.

FIGURE S3 Sample recordings of spontaneous excitatory postsynaptic currents (sEPSCs) recorded at −70 mV using the whole-cell patch-clamp technique from pyramidal neurons of minimal spiking region (MIN) and maximal spiking region (MAX) samples of both EO and late-onset (LO) patients with FCD.

TABLE S1 Comparison of characteristics of spontaneous inhibitory postsynaptic currents (sIPSCs) and miniature IPSCs (mIPSCs) in MIN and MAX samples of patients with FCD.
