# Supplementary figures and images for: GABAA Receptor-Mediated Epileptogenicity in Focal Cortical Dysplasia (FCD) Depends on Age at Epilepsy Onset
Source: Front Cell Neurosci. 2020 Sep 30;14:562811. doi: 10.3389/fncel.2020.562811 (PMC7556289; doi:10.3389/fncel.2020.562811)

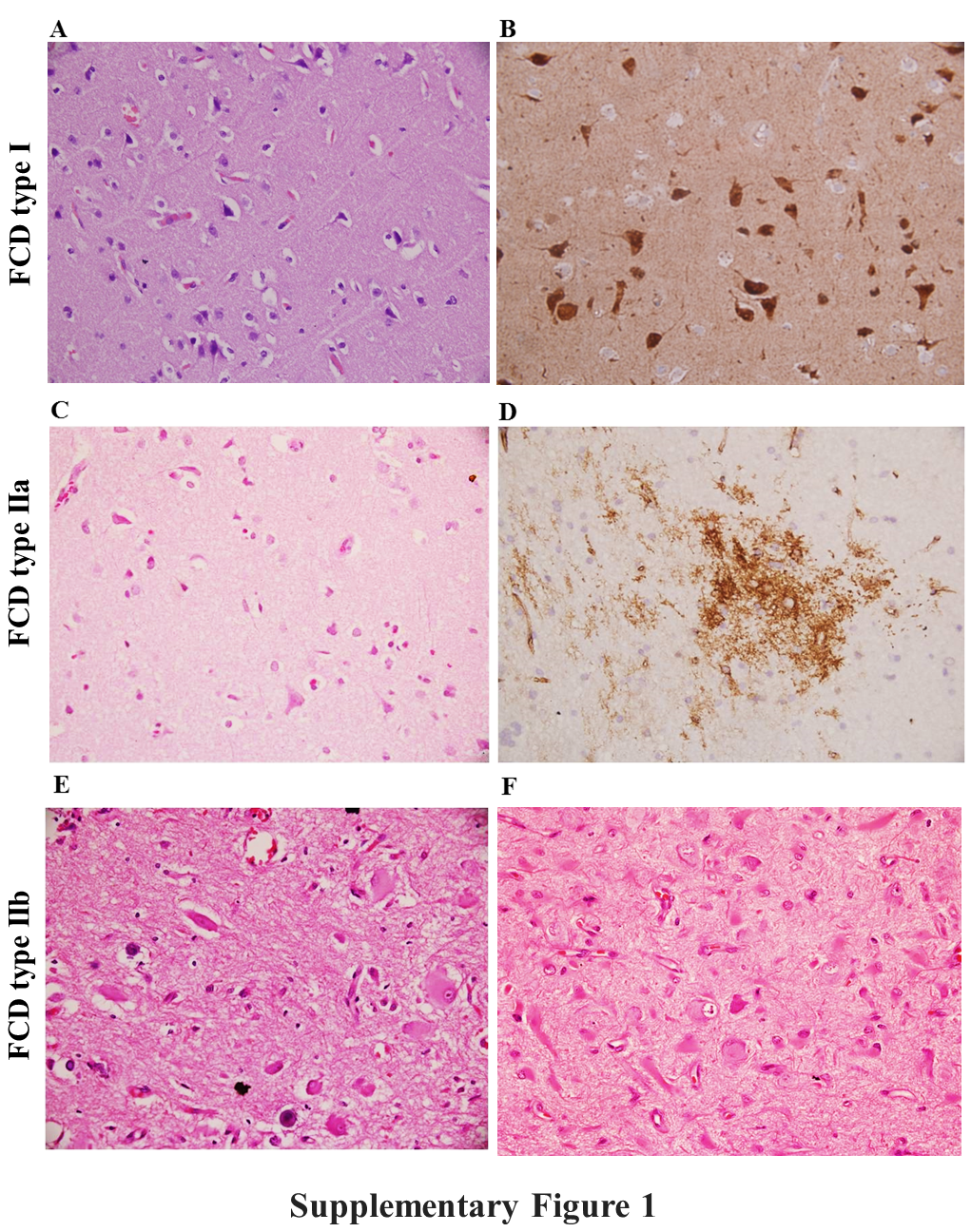

Supplement: FIGURE S1 — Histology revealed abnormal cytoarchitecture in resected brain specimens obtained from patients with focal cortical dysplasia (FCD). (A) H&E staining of FCD type I sections showing loss of laminar architecture of the cerebral cortex, the haphazard arrangement of the neurons (×400). (B) NeuN immunohistochemistry shows abnormal neurons express immunopositivity for anti-NeuN antibody (magnification 400×). (C) H&E staining of FCD type IIa sections showing loss of laminar architecture (×400). (D) CD34 immunohistochemistry shows some of these clustered abnormal neurons express bush like immunopositivity For CD34 (magnification 400×). (E,F) H&E staining of FCD type IIb sections showing neuromegaly, loss of laminar architecture and larger neurons with abundant eosinophilic cytoplasm (glassy neurons; magnification 400×). [file Image_1.tif]

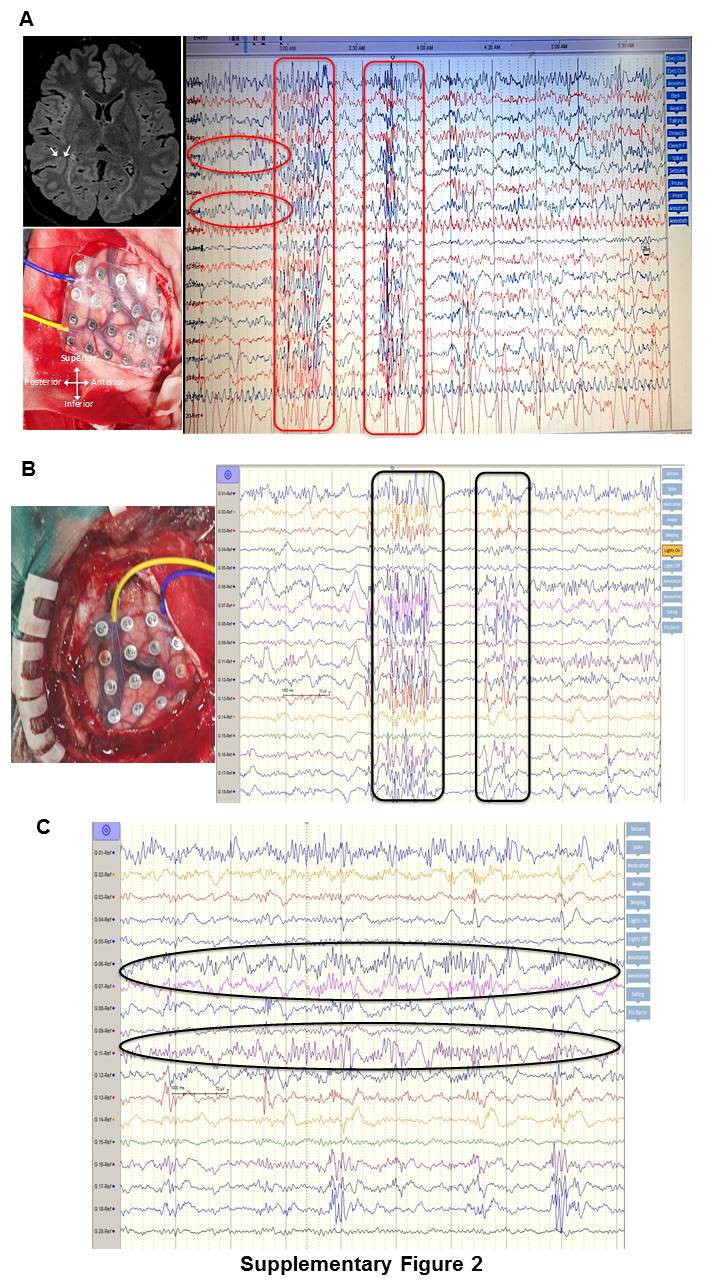

Supplement: FIGURE S2 — Representative electrocorticography (ECoG) of a 13-year-old male patient with early-onset (EO) FCD. (A) Top left panel displays MRI brain axial section FLAIR sequence showing right posterior superior temporal gyrus bottom of sulcus dysplasia. Intraoperative picture (bottom left panel) showing a 4 × 5 ECoG grid placed on the temporoparietal region. The right panel shows preoperative ECoG recording (sensitivity-300 μv/cm & high cut-70 Hz) with poly spike bursts beginning in the leads 8 and 5 (red ellipse) to involve all the leads producing multiple brief burst like pattern (red rectangles) against a fast background representing grade 3 (Mathern et al., 2000; Tripathi et al., 2010). (B) ECoG grid was placed over the resection cavity, following the first trans-sulcal resection of the dysplasia (left panel). ECoG tracings (right panel) showing persistent spikes (grade 3) occurring in brief bursts (black rectangles). (C) ECoG recording following the extension of resection (second resection) to the overlying temporal and partly parietal cortex surrounding the previous resection cavity. The ECoG tracing improved from grade-3 to grade-2. [file Image_2.tif]
