# Supplementary material for: GABAA Receptor-Mediated Epileptogenicity in Focal Cortical Dysplasia (FCD) Depends on Age at Epilepsy Onset
Source: Front Cell Neurosci. 2020 Sep 30;14:562811. doi: 10.3389/fncel.2020.562811 (PMC7556289; doi:10.3389/fncel.2020.562811)
Supplement: TABLE S1 — Comparison of characteristics of spontaneous inhibitory postsynaptic currents (sIPSCs) and miniature IPSCs (mIPSCs) in MIN and MAX samples of patients with FCD. [file Table_1.docx]

**Supplementary Table S1: Comparison of characteristics of sIPSCs and mIPSCs in MIN and MAX samples of patients with FCD**: Kinetic parameters of sIPSC and mIPSC recorded from pyramidal neurons in MIN and MAX samples obtained from patients with FCD. Data are presented as mean ± SEM of results obtained from 24 neurons each from MIN and MAX samples of 24 patients with FCD. *, p < 0.05; **, p < 0.01 compared to MIN samples according to one-way ANOVA followed by Dunnett post-hoc test.

| **Sample** | **Frequency (Hz)** | **Amplitude (pA)** | **Rise Time 10 to 90% (ms)** | **τ_d_ (ms)** |
| --- | --- | --- | --- | --- |
| **sIPSC** | | | | |
| **MAX** | **3.0 ± 0.4**** | **27.1 ± 3.5**** | **1.9 ± 0.2** | **43.9 ± 3.5** |
| **MIN** | **2.5 ± 0.3** | **22.7 ± 2.0** | **1.9 ± 0.3** | **31.2 ± 3.0** |
| **mIPSC** | | | | |
| **MAX** | **1.2 ± 0.2**** | **15.3 ± 1.5*** | **1.84 ± 0.3** | **39.5 ± 2.7*** |
| **MIN** | **0.9 ± 0.1** | **12.8 ± 1.2** | **1.79 ± 0.4** | **37.7 ± 2.9** |
